# Determining Surface Shape of Translucent Objects with the Combination of Laser-Beam-Based Structured Light and Polarization Technique

**DOI:** 10.3390/s21196587

**Published:** 2021-10-01

**Authors:** Bingquan Chen, Peng Shi, Yanhua Wang, Yongze Xu, Hongyang Ma, Ruirong Wang, Chunhong Zheng, Pengcheng Chu

**Affiliations:** 1 Research Center for Quantum Optics and Quantum Communication, School of Science, Qingdao University of Technology, Qingdao 266525, China; chen_bingquan@yeah.net (B.C.); wangyanhua003@163.com (Y.W.); xuyongze0302@163.com (Y.X.); mahongyang@qut.edu.cn (H.M.); zhengchunhong@qut.edu.cn (C.Z.); kyois@126.com (P.C.); 2 Office of Laboratory Management, Qingdao Agricultural University, Qingdao 266109, China; 199201019@qau.edu.cn

**Keywords:** 3D reconstruction, translucent objects, coherent projection, polarization, optical filtering, Fourier transform

## Abstract

In this study, we focus on the 3D surface measurement and reconstruction of translucent objects. The proposed approach of surface-shape determination of translucent objects is based on the combination of the projected laser-beam-based sinusoidal structured light and the polarization technique. The theoretical analyses are rigorously completed in this work, including the formation, propagation, and physical features of the generated sinusoidal signal by the designed optical system, the reflection and transmission of the projected monochromatic fringe pattern on the surface of the translucent object, and the formation and the separation of the direct-reflection and the global components of the surface radiance of the observed object. The results of experimental investigation designed in accordance with our theoretical analyses have confirmed that accurate reconstructions can be obtained using the one-shot measurement based on the proposed approach of this study and Fourier transform profilometry, while the monochromaticity and the linearly-polarized characteristic of the projected sinusoidal signal can be utilized by using a polarizer and an optical filter simultaneously for removing the global component, i.e., the noised signal contributed by multiply-scattered photons and the background illuminance in the frame of our approach. Moreover, this study has also revealed that the developed method is capable of getting accurate measurements and reconstructions of translucent objects when the background illumination exists, which has been considered as a challenging issue for 3D surface measurement and reconstruction of translucent objects.

## 1. Introduction

It has been attractive but extremely challenging for the in situ measurement and accurate reconstruction of translucent objects. Generally speaking, the pure direct reflection can be used for determining the 3D shape of a translucent object. However, the physical feature of translucent objects determines that the signal of the direct reflection is usually quite weak, while most parts of the illumination will go through the interface and undergo the multiple scattering processes. The surface reflectivity of the translucent object could be lower less than 5%, such as an object made of silicone rubber with the real part of the refractive index being $n_{r}=1.5$. The optical triangular method has been considered as a popular and promising technique in this area. However, as discussed by Holroyd and Lawrence [1], it would be difficult to carry out 3D shape measurement of translucent objects using optical triangular methods, i.e., the structured-light-illumination methods, due to the subsurface scattering brings uncertainties to the reflection measurements at the object surface. The optical triangular method is simply based on the assumption that the direct reflection takes place at the object surface, which might be violated for measuring the translucent objects.

The basic approach of previous investigations for measuring the translucent objects using optical triangular methods is isolating the direct reflectance at the object surface [1,2,3,4]. The method of separating the direct and global components was initially investigated by Nayar et al. [5], which has been cited, discussed, and adopted by other works [6,7]. In the approach of Nayar et al. the global lighting effect was considered as a constant if the projected high-frequency pattern was employed, which allowed for efficient separation of the observed light intensities into direct and global light [5]. Generally speaking, it is difficult to separate the direct and global components, and the separation technique developed by Nayar et al. basically needs a lot of images, which may also be affected by the noises existed in the whole process [5]. To this end, Lockerman and co-authors proposed a method using three directions of projection [8].

The study of polarization-difference imaging [9,10] and the method combining phase-shifting and polarization filtering [11] are considered as promising approach for improving 3D surface measurement of translucent objects. Recently, Xu and co-authors proposed an approach for 3D shape measurement of translucent objects based on phase-shifting fringe projection profilometry [12]. Even though phase-shifting method can be considered to perform the separation of the direct and global components and the 3D surface measurement simultaneously [5,11], measurements using phase-shifting method certainly have a strict requirement of surrounding environment, such as that there should not be background illumination and environmental vibration. For reducing the measuring error in 3D scans of translucent objects using illuminated fringe patterns [13], a number of novel investigations have been carried out, such as the approaches based on multiple scattering [14], the photometric stereo method for measuring optically-thick translucent objects [15], the Monte Carlo simulation [16], and the Fourier single-pixel imaging technique [17]. For the binary boundary methods, exclusively high or low-frequency pattern schemes were considered robust against different global illumination effects [18]. Note that, edges in images of translucent objects are very different from edges in images of opaque objects [19], since the internal scattering within the translucent object can create a variety of image effects, basically depending on the shape and material of the observed object as well as the optical configuration of the measurement.

The existing approaches of measuring translucent objects using the optical triangular method are basically based on the phase-shifting technique and the projection of visible-light fringe patterns generated by the DMD-chip-based digital-light-processing (DLP) projector. However, the phase-shifting technique does have a rigorous requirement of the observed object and the environment, i.e., there is no environmental vibration and the observed object has to remain still during the measuring time. As far as the structured light generated by DLP projector, the intensity of projected fringe pattern is affected by the Gamma effect and defocusing, which can result in inaccuracy of image processing and reconstruction. Moreover, for using projections of visible-light fringe patterns, it is basically impossible to carry out measurements under background illumination and obtain reconstructions with better accuracy. Thus, it might be difficult to fulfill the outdoor and/or in situ measurements of translucent objects using the optical triangular method together with the phase-shifting technique and the projection of DLP-based visible-light fringe patterns due to the exist of environmental vibration and background illumination.

Therefore, for obtaining an improved solution to the challenging of separating the direct and global components of measuring the translucent object as well as overcoming the drawbacks of the phase-shifting technique with the DLP-based fringe patterns, we suggest and investigate a new method using the monochromatic structured light, techniques of optical filtering and polarization, and the image-processing algorithm based on one-shot Fourier transform profilometry.

## 2. Theoretical Analysis and Mathematical Description of the Proposed Method

This section presents a discussion of the physical process of the surface-shape measurement and reconstruction of a translucent object using sinusoidal fringe pattern generated by the developed optical system, which basically contains the theoretical analyses and mathematical descriptions of the generation and propagation of the sinusoidal fringe pattern in the air, the projection and reflection of the sinusoidal fringe pattern on the surface of the object being observed, and the multiple-scattering process of the transmitted sinusoidal optical signal under the surface of the translucent object and its contribution to the radiance measured by the CCD camera.

### 2.1. Generation and Propagation of the Sinusoidal Fringe Signal

**Figure 1:**
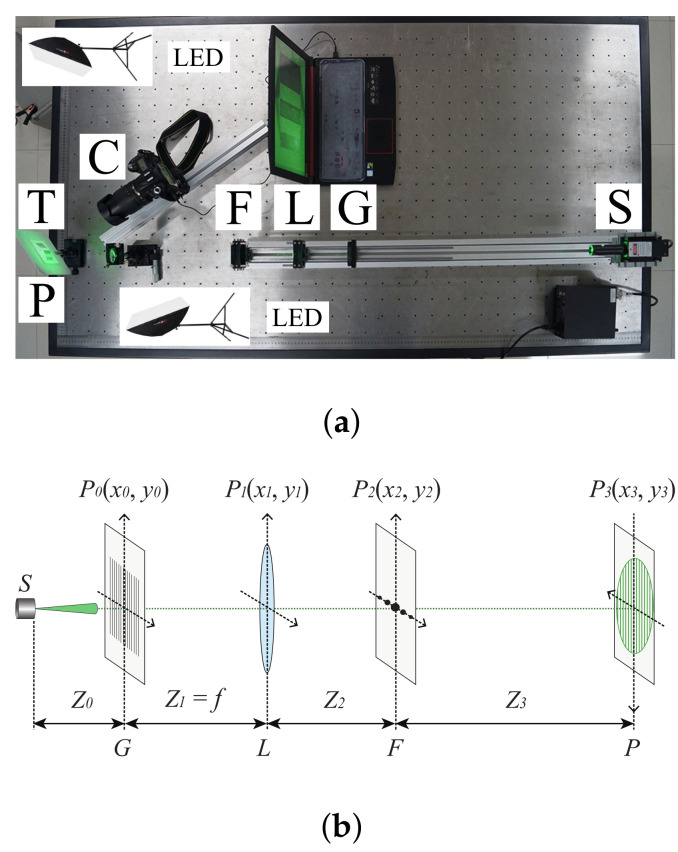
The developed the laser-beam-based optical system. (**a**) Experimental setup. *S*: CW laser source; *G*: grating; *L*: Fourier-transform positive lens; *F*: spatial-frequency filter; *P*: observation plane; *T*: target on the observation plane; *C*: CCD Camera connected to a computer; LED: LED light for accurately adjusting the background illuminance. (**b**) Sketch of the sinusoidal optical signal generator.

As we discussed in the section of introduction, the proposed method of the surface-shape measurement and reconstruction of a translucent object is based on the combination of the projection of the monochromatic structured light, techniques of optical filtering and polarization, and the image-processing algorithm of Fourier transform profilometry. Profilometry based on either Fourier-transform approach or phase-shifting method relies on the projection of sinusoidal fringe patterns. The widely-used sinusoidal fringe pattern generated using DLP projector is non-monochromatic structured signal, which indicates that it is impossible to employ the techniques of optical filtering and polarization when the projected fringe pattern is generated using DLP projector. For the purpose of generating monochromatic, high contrast, and truly sinusoidal fringe patterns, we have designed and developed the laser-beam-based optical system as shown in Figure 1a. The developed optical system consists of the following parts: a CW laser source *S* with the wavelength λ=532 nm, a rectangular grating *G*, a Fourier-transform positive lens *L* with focal length *f*, an adjustable spatial-frequency filter *F*, the observation plane *P* where the target *T* is placed, and a CCD camera *C* being connected to the computer. As sketched in Figure 1b, the fundamental components of the sinusoidal optical signal generator include a CW laser source *S*, a grating positioned at the plane $P_{0}$, a Fourier-transform positive lens at the plane $P_{1}$, an adjustable spatial-frequency filter located at the plane $P_{2}$, and the observation plane is set at the plane $P_{3}$.

To analyze the generation and propagation of the sinusoidal fringe signal, we start with the filed from a CW point laser source *S*, U(r), in the form [20]

(1) $$\begin{array}{c} U\left(r\right)=\frac{A}{r}e^{ikr}, \end{array}$$

where *k* is the wave number in the homogeneous background medium, i.e., the air in this study, *A* is a complex constant related to the laser power, and $r=\sqrt{x^{2}+y^{2}+z^{2}}$. Taking A=1 for simplicity and using the paraxial approximation, the field $U_{0}\left(x_{0},y_{0}\right)$ behind the grating *G* at the plane $P_{0}$ is given by [21]

(2) $$\begin{array}{c} U_{0}\left(x_{0},y_{0}\right)=\frac{1}{Z_{0}}e^{ikZ_{0}}e^{i\frac{k}{2Z_{0}}\left(x_{0}^{2}+y_{0}^{2}\right)}t_{0}\left(x_{0},y_{0}\right), \end{array}$$

where $x_{0}$ and $y_{0}$ are the coordinate variables of $P_{0}$, $Z_{0}$ is the distance between the laser source *S* and grating *G*, and $t_{0}\left(x_{0},y_{0}\right)$ the transmission function of *G*. Considering that *G* is one-dimensional, $t_{0}\left(x_{0},y_{0}\right)$ can be described using [22]

(3) $$\begin{array}{c} t_{0}\left(x_{0},y_{0}\right)=t_{0}\left(x_{0}\right)=\left[\mathrm{rect}\left(\frac{x_{0}}{a}\right)*\frac{1}{d}\mathrm{comb}\left(\frac{x_{0}}{d}\right)\right]\mathrm{circ}\left(\frac{2|x_{0}|}{H}\right), \end{array}$$

where *a* and *d* are the optical parameters of grating *G*. *H* is the grating width, which is assumed, for the sake of simplicity, to be the diameter of the illumination spot of laser.

Since the propagation of field $U_{0}\left(x_{0},y_{0}\right)$ from plane $P_{0}$ to plane $P_{1}$ is within Fresnel region, the field $U_{1}^{\prime }\left(x_{1},y_{1}\right)$ in front of the lens *L* at plane $P_{1}$ can be written as

(4) $$\begin{array}{c} U_{1}^{\prime }\left(x_{1},y_{1}\right)=C_{1}\int_{\Sigma_{0}}U_{0}\left(x_{0},y_{0}\right)e^{i\frac{k}{2Z_{1}}\left[{\left(x_{1}-x_{0}\right)}^{2}+{\left(y_{1}-y_{0}\right)}^{2}\right)]}dx_{0}dy_{0}, \end{array}$$

where $C_{1}=\frac{1}{i\lambda Z_{1}}e^{ikZ_{1}}$ is a complex constant, and $Z_{1}$ is the distance between the planes $P_{0}$ and $P_{1}$. The field $U_{1}\left(x_{1},y_{1}\right)$ after the lens *L* at plane $P_{1}$ can be represented as

(5) $$\begin{array}{c} U_{1}\left(x_{1},y_{1}\right)=U_{1}^{\prime }\left(x_{1},y_{1}\right)t_{L}\left(x_{1},y_{1}\right)=U_{1}^{\prime }\left(x_{1},y_{1}\right)e^{-i\frac{k}{2f}\left(x_{1}^{2}+y_{1}^{2}\right)}, \end{array}$$

where $t_{L}\left(x_{1},y_{1}\right)=e^{-i\frac{k}{2f}\left(x_{1}^{2}+y_{1}^{2}\right)}$ denotes the transmission function of the lens *L*.

The propagating field $U_{1}\left(x_{1},y_{1}\right)$ at plane $P_{1}$ to $U_{2}^{\prime }\left(x_{2},y_{2}\right)$ at plane $P_{2}$ can be accurately computed using Fresnel diffraction. $U_{2}^{\prime }\left(x_{2},y_{2}\right)$ denotes the field in front of the spatial-frequency filter *F*, and is given by

(6) $$\begin{array}{c} U_{2}^{\prime }\left(x_{2},y_{2}\right)=C_{2}\int_{\Sigma_{1}}U_{1}\left(x_{1},y_{1}\right)e^{i\frac{k}{2Z_{2}}\left[{\left(x_{2}-x_{1}\right)}^{2}+{\left(y_{2}-y_{1}\right)}^{2}\right)]}dx_{1}dy_{1}, \end{array}$$

where $C_{2}=\frac{1}{i\lambda Z_{2}}e^{ikZ_{2}}$ is a complex constant, and $Z_{2}$ is the distance between the planes $P_{1}$ and $P_{2}$. Combining Equations (2)–(6) as well as the expression of $t_{L}\left(x_{1},y_{1}\right)$, we have

(7) $$\begin{array}{c} U_{2}^{\prime }\left(\omega \right)=\frac{aC_{3}}{d}\sum_{m=-\infty }^{\infty }\mathrm{sinc}\left(\frac{am}{d}\right)\frac{J_{1}\left[\pi H\left(\omega -m/d\right)\right]}{\omega -m/d}, \end{array}$$

where $J_{1}\left(\omega \right)$ is the Bessel function of the first kind, $\omega =2\pi f_{x}$ with the spatial frequency $f_{x}$ at plane $P_{2}$ defined by $f_{x}=\frac{x_{2}}{\lambda f}$, and $C_{3}$ is a complex constant given by $C_{3}=\frac{1}{\lambda fZ_{0}}e^{i[k\left(Z_{0}+Z_{1}+Z_{2}\right)-\pi /2]}$.

The role of the adjustable spatial-frequency filter *F* at plane $P_{2}$ is to select ±m-th order spectrum described in Equation (7), and let them to pass through it, and here we take m=1. We then get the field $U_{2}\left(x_{2},y_{2}\right)$ right behind the filter *F* in the following form

(8) $$\begin{array}{c} U_{2}\left(x_{2},y_{2}\right)=U_{2}\left(\omega \right)=U_{2}^{\prime }\left(\omega \right){|}_{m=\pm 1}=\frac{aC_{1}}{d}\mathrm{sinc}\left(\frac{a}{d}\right)\{\frac{J_{1}\left[\pi H\left(\omega -1/d\right)\right]}{\omega -1/d}+\frac{J_{1}\left[\pi H\left(\omega +1/d\right)\right]}{\omega +1/d}\}. \end{array}$$

The propagation of field $U_{2}\left(x_{2},y_{2}\right)$ from plane $P_{2}$ to $P_{3}$ can also be analyzed using Fresnel diffraction, which let the field $U_{3}\left(x_{3},y_{3}\right)$ at plane $P_{3}$ to be given by

(9) $$\begin{array}{c} U_{3}\left(x_{3},y_{3}\right)=C_{4}\int_{\Sigma_{2}}U_{2}\left(x_{2},y_{2}\right)e^{i\frac{k}{2Z_{3}}\left[{\left(x_{3}-x_{2}\right)}^{2}+{\left(y_{3}-y_{2}\right)}^{2}\right)]}dx_{2}dy_{2}, \end{array}$$

where $C_{4}=\frac{1}{i\lambda Z_{3}}e^{ikZ_{3}}$ is another complex constant, and $Z_{3}$ is the distance between the planes $P_{2}$ and $P_{3}$. Considering that the size of $\Sigma_{2}\left(x_{2},y_{2}\right)$ (≤4 mm in diameter) is much less than that of $\Sigma_{3}\left(x_{3},y_{3}\right)$, i.e., the spot size of the sinusoidal fringe pattern (≥100 mm in diameter), we take an approximation that $\lambda f\left(f_{x}^{2}+f_{y}^{2}\right)\ll 2\left(f_{x}x_{3}+f_{y}y_{3}\right)$ for further derivation of Equation (9). Thus, Equation (9) becomes

(10) $$\begin{array}{c} U_{3}\left(x_{3},y_{3}\right)=C_{5}\int_{\Sigma_{2}}U_{2}\left(f_{x},f_{y}\right)e^{i2\pi (f_{x}\frac{fx_{3}}{Z_{3}}+f_{y}\frac{fy_{3}}{Z_{3}})}df_{x}df_{y}=C_{5}F^{-1}\left\{U_{2}\left(f_{x},f_{y}\right)\right\}, \end{array}$$

where the complex parameter $C_{5}$ is given by $C_{5}=\frac{\lambda f^{2}}{Z_{3}}e^{i(kZ_{3}-\pi /2)}e^{i\frac{k}{2Z_{3}}\left(x_{3}^{2}+y_{3}^{2}\right)}$.

Equation (10) indicates that the field at the observation plane $P_{3}$ is an inverse Fourier transform of the field output from the spatial-frequency filter *F*. Combining Equations (8) and (10), we have

(11) $$\begin{array}{c} U_{3}\left(x_{3}\right)=C_{6}\cdot \mathrm{circ}\left(\frac{2f|x_{3}|}{HZ_{3}}\right)\cdot \cos\left(\frac{2\pi fx_{3}}{dZ_{3}}\right), \end{array}$$

(12) $$\begin{array}{c} C_{6}=\frac{2f}{Z_{0}Z_{3}\pi }\sin\left(\frac{a\pi }{d}\right)e^{i[k\left(Z_{0}+Z_{1}+Z_{2}+Z_{3}\right)-\pi ]}e^{i\frac{k}{2Z_{3}}\left(x_{3}^{2}+y_{3}^{2}\right)}. \end{array}$$

As indicated in Equation (12), $C_{6}$ is obviously a complex constant for determined spatial distances $Z_{0}$ and $Z_{3}$, and the modulus of $C_{6}$ represents the amplitude of the generated sinusoidal signal. We see that the fringe intensity will decrease as $Z_{3}$ increases since *f* and $Z_{0}$ are generally parameters with fixed values. The part of $\mathrm{circ}\left(\frac{2f|x_{3}|}{HZ_{3}}\right)$ in Equation (11) results in a definition of the range of the fringe pattern with a circle of radius $\frac{HZ_{3}}{2f}$. The term of $\cos\left(\frac{2\pi fx_{3}}{dZ_{3}}\right)$ is the key part of output of the designed optical system, i.e., the expression of a sinusoidal curve.

### 2.2. Reflection and Transmission of the Projected Sinusoidal Fringe Signal on the Surface of the Translucent Object

**Figure 2:**
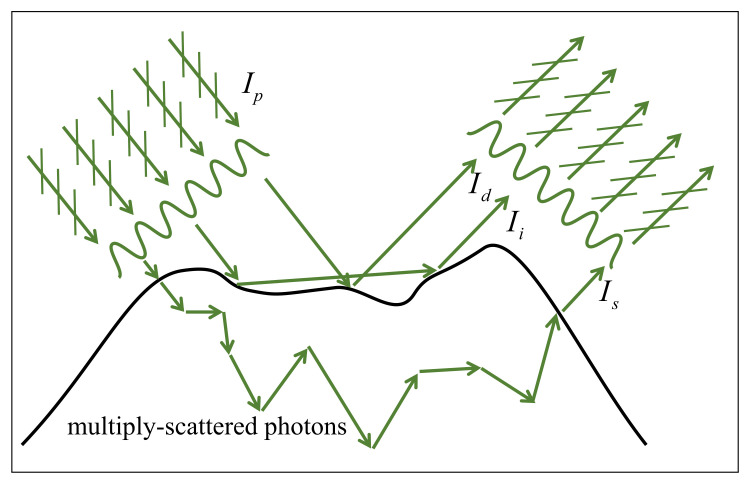
Diagram of the projection, reflection, and transmission of the sinusoidal optical signal on the surface of a translucent object. $I_{p}$ is the intensity of the projected fringe pattern; $I_{d}$ stands for the directly-reflected radiance; $I_{i}$ represents the radiance of interreflection; $I_{s}$ denotes the contribution from multiply-scattered photons.

The proposed method of surface-shape measurement of translucent objects relies on the projection of laser-beam-based structured light, i.e., the monochromatic, high contrast, and truly sinusoidal optical signal described using Equation (11). Note that the projected fringe pattern described by Equation (11) is not affected by the defocusing issue, while the widely-used DLP projector does have the featured problem of defocusing. $U_{3}\left(x_{3}\right)$ given in Equation (11) is coherent and linearly polarized before reaching the surface of the translucent object. As shown in Figure 2, the projected sinusoidal fringe signal undergoes the reflection and transmission on the surface of a translucent object and multiple scattering beneath the surface.

We see from Figure 2 that the total radiance measured by the detector will basically consist of $I_{d}$, the directly-reflected radiance, $I_{i}$, the radiance of interreflection, $I_{s}$, the contribution from multiply-scattered photons, and $I_{b}$, the contribution of background illuminance. In the proposed method of this work, $I_{d}$ is the only component that will be used for surface-shape reconstruction of the translucent object being observed, which will raise a challenging issue of separating $I_{d}$ from the rest parts, i.e., $I_{i}$, $I_{s}$ and $I_{b}$, of the totally-measured radiance. The physical characteristics of $I_{d}$, $I_{i}$, $I_{s}$ and $I_{b}$ can be summarized as follows.

(i) $I_{d}$: The directly-reflected radiance, $I_{d}$, is the reflected portion of the projected fringe pattern, which belongs to diffuse reflection. Since the surface property of the observed object is varying, some fraction of the direct reflection might be depolarized. When the energy of projection is fixed, $I_{d}$ basically depends on the real part of refraction index of the observed object in accordance with the Fresnel’s Equations for reflection and transmission. For the translucent objects used in this work, the real part of refraction index $n_{r}$ = 1.4∼1.5, which indicates that $I_{d}$ is only about 3∼4% of $I_{p}$, the intensity of the projected fringe pattern. However, $I_{d}$ is the only part that will be picked up and used for reconstruction of the translucent objects being observed.

(ii) $I_{i}$: Based on Fresnel’s Equations we can conclude the following two points: First, the polarization direction of $I_{i}$ is different from that of $I_{d}$. Second, as shown in Figure 2, the dominant part of $I_{i}$ is the inter-reflections of the incident signal. However, the magnitude of $I_{i}$ is lower than the incident signal by about two orders due to the reason that the surface reflectivity of the translucent object being observed is *R* = 2.8∼5.3% with an assumption that the real part of the refractive index of the translucent object is in the range of $n_{r}$ = 1.4∼1.6 [23,24].

(iii) $I_{s}$: The contribution to the measured radiance from multiply-scattered photons, $I_{s}$, is unpolarized. Except $I_{d}$, the rest part of the projected signal will transmit the surface, undergo a multiple-scattering process within the translucent object, and exit from the surface of the translucent object. $I_{s}$ can be estimated using diffusion approximation [25,26]. For calculating $I_{s}$, the successful approach is a one-dimensional model based on radiative transfer theory [26]. Thus, we believe that it would be difficult to complete 3D reconstruction based on one-dimensional model of $I_{s}$ calculation.

(iv) $I_{b}$: The background illuminance, $I_{b}$, is unpolarized and polychromatic. It should be noted that if the projected signal is polychromatic, the surface measurement of the translucent object should be suggested to be carried out in a dark environment to keep $I_{b}=0$. Otherwise, it would be very difficult or extremely time-consuming to separate $I_{d}$ from $I_{b}$.

It should be noted that, for steady projection of the sinusoidal fringe pattern on a static translucent object being observed, $I_{i}$ and $I_{s}$ are time-independent, while $I_{b}$ is time-dependent since the background illumination is always varying. However, for measuring a dynamic translucent object, all of the values of $I_{i}$, $I_{s}$, and $I_{b}$ are time-dependent. However, the time-dependent values of $I_{i}$, $I_{s}$, and $I_{b}$ have no effects on the developed method of this investigation, which is one of the few important issues that may result in the expectation of applying the developed method to the measurement of dynamic translucent objects.

### 2.3. Separating $I_{d}$ from $I_{g}$

Referring to the general definition of the global component previously suggested by Nayar and co-workers [5], the total radiance measured by the detector is given by

(13) $$\begin{array}{c} I=I_{d}+I_{g}, \end{array}$$

where $I_{d}$ and $I_{g}$ stands for the direct and the global components, respectively. Based on the proposed model discussed above, the global component $I_{g}$ contains the following three parts

(14) $$\begin{array}{c} I_{g}=I_{b}+I_{i}+I_{s}, \end{array}$$

where $I_{b}$ is the background illumination, $I_{i}$ represents the radiance of inter-reflection, and $I_{s}$ stands for the contribution from multiply-scattered photons.

Therefore, if the proposed methods can work for removing the three components of $I_{g}$, i.e., $I_{b}$, $I_{i}$, and $I_{s}$, the expected signal of $I_{d}$ can then be successfully measured, which is critically important for the 3D surface measurement and reconstruction of translucent objects.

#### 2.3.1. Removing the Effect of $I_{b}$ Using an Optical Filter

The component $I_{b}$ related to background illumination is one important issue for in situ profilometry based on either Fourier transform or phase-shifting method, which has been the main reason that most of previous investigations have carried out experimental study in a dark environment for keeping $I_{b}=0$.

In the proposed approach of this work, we are using the laser-beam-based monochromatic structured light at 532 nm as the projecting fringe patterns, which makes it possible to employ the optical filtering technique in the process of measurement. In practice, an optical bandpass filter centered at 532 nm is employed for removing the effects of background illuminations in the experimental setup of this investigation.

#### 2.3.2. Eliminating the Effect of $I_{i}$ and $I_{s}$ Using a Polarizer

There have been some efforts that 3D reconstructions are based on the multiply-scattered photons, i.e., $I_{s}$, such as the work by Ohtani et al. [14]. However, as we discussed above, the theoretical modeling and computation based on radiative transfer and diffusion theory for the reconstruction of the observed object must be difficult, since only one-dimensional model has been theoretically developed and experimentally validated [26], while it is almost impossible to describe the observed objects using one-dimensional model. In the work of Umeyama and Godin, a method of separating the diffuse and specular components of surface reflection using polarization as well as statistical analysis of images was proposed, but it was only validated for measuring the opaque objects [27].

In the developed method of this work, the desired useful signal is $I_{d}$, and $I_{s}$ is then taken as noised signal to be eliminated. The component $I_{s}$ will be eliminated via employing polarization technique, which can be ensured since the incident monochromatic structured light and reflected signal from the surface of the translucent objects being observed are linearly polarized, and the part of signal composing multiply-scattered photons is completely unpolarized. When carrying out the practical measurement, a polarizer is mounted on the camera to carry out the measurement of observed object with projected fringe patterns generated using our designed laser-beam-based optical system as sketched in Figure 1.

As far as $I_{i}$ is concerned, since the magnitude of $I_{i}$ is much lower than $I_{d}$ and the polarization direction of $I_{i}$ is different from that of $I_{d}$ as discussed above, the elimination of $I_{i}$ can be accomplished when a polarizer is employed.

The angle of rotation of the polarizer is needed to be carefully controlled before taking the image or measurement of the object being observed. Note that, when having the same object and the same direction of fringe projection, the rotation of the polarizer is basically not necessary based on the discussion above, for the reason that the direction of polarization of $I_{d}$ should be unchanged. For measuring another object or having a new direction of fringe projection, the direction of polarization of $I_{d}$ will certainly be different, which will result in the requirement of rotation of the polarizer as a necessary procedure during the measurement.

## 3. Technical Analysis of Measurement and Reconstruction

In this investigation, the experimental setup for carrying out surface measurements of translucent objects is based on Figure 1a. From Figure 1a, we see that the parts *S*, *G*, *L*, and *F* are basically the main components of the sinusoidal optical signal generator. The laser-beam-based optical system employs a CW-laser source *S* with the wavelength λ=532 nm, maximum output power of 200 mW, and a small divergence angle (≤1.5${}^{\circ }$). A rectangular grating *G* illuminated by the laser beam is placed at a front focal point of a positive lens *L*. On the conjugate plane of *S*, an adjustable spatial-frequency filter *F* is set, which is considered that the part *F* plays a critical role in this system. For this spatial-frequency filter, a V-shape aperture with a width of 0.5 mm is carved with high precision, we can then choose and allow the selected order of the spatial frequency to pass through. The output of a sinusoidal fringe pattern can then be observed on the observation plane *P*, as indicated in Figure 1a. The design of this adjustable spatial-frequency filter *F* containing three accurately cut slits is actually based on our previous work of measuring modulation transfer function [28] and other recent studies on 3D surface measurements [29,30].

Note that the position of observation plane *P* is arbitrary, e.g., from few-ten centimeters to few-ten meters, since the projected fringe pattern is not affected by the defocusing issue as we discussed above. At any value of $Z_{3}$ in Figure 1b, we have the monochromatic, high contrast, and truly sinusoidal optical signal described using Equation (11), while the only difference is the spatial frequency of the projected fringe pattern as well as the maximum intensity of the fringe that is inversely proportional to the square of $Z_{3}$.

With the proposed approach of this investigation, accurate reconstructions of translucent objects can be achieved for background illuminance up to *E* = 3000 Lux, which is realized using an optical bandpass filter centered at 532 nm with FWHM being 10 nm for removing the effects of background illuminations. The background illuminance *E* was adjusted using LED lights, and it was measured using a digital luxmeter placed beside the observation plane *P*. Meanwhile, as discussed above, a polarizer is employed for eliminating the part of signal composing the inter-reflected photons on the surface of the translucent object and the multiply-scattered photons exiting from the subsurface of the translucent object. Both the optical bandpass filter and the polarizer are mounted on the measuring camera.

Reconstructions of the observed translucent objects will be processed and obtained using those measurements based on Fourier transform profilometry (FTP). The method of FTP was initiated by Takeda and Mutoh [31], and it has been applied and improved in many investigations of 3D surface measurement [32,33,34,35]. However, applications of FTP have been limited to the situations with ideal conditions, while such necessary conditions may include that the surface of the measuring object must be opaque, the surface is highly reflective and diffuse, and there should be no background illumination. Thus, the quality of the measurement of surface reflectance provides a necessary basis for developing an image-processing algorithm for the retrievals of phase and 3D-surface height of the observed translucent object. The image-processing algorithm of the proposed approach in this work is based on FTP with a typical triangulation framework of 3D surface measurement, while the general framework of 3D surface measurement can be found in previous works published by Takeda and Mutoh [31], and by Maurel and co-workers [32].

## 4. Experimental Results and Discussion

**Figure 3:**
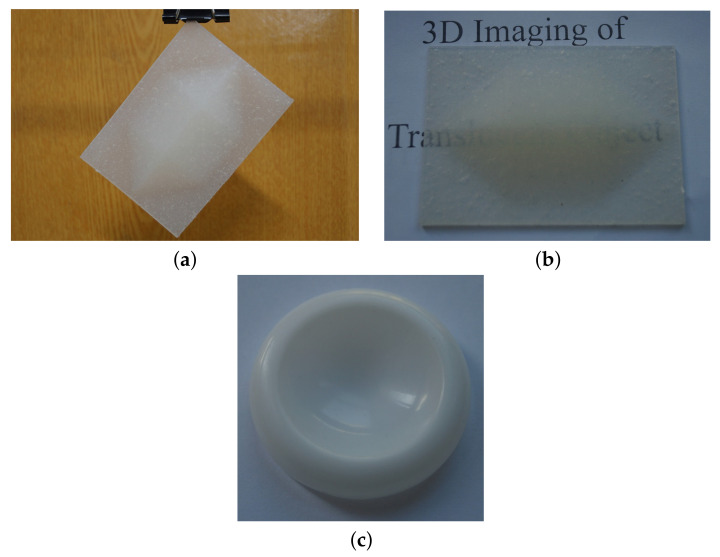
Original photos of selected translucent objects being measured. (**a**) Heptahedron-shape vulcanized silicone rubber. (**b**) The same object shown in (**a**) on white paper with printed letters. (**c**) Translucent plastic cap.

There are two selected objects being measured in this study, including (i) a heptahedron-shape target similar to an elongated triangular dipyramid that was made of translucent room-temperature-vulcanized silicone rubber via two-component addition molding in the lab as shown in Figure 3a,b, and (ii) a translucent plastic cap made of polypropylene as shown in Figure 3c. The real part of refractive index of vulcanized silicone rubber at 532 nm is about $n_{r}=1.41$ [23], and the real part of the refractive index of the plastic cap is about $n_{r}$ = 1.47∼1.49 [24]. The designed heptahedron-shape target has a set of given geometrical parameters, which is necessary and important for validation study and determining the accuracy of the proposed approach.

### 4.1. Measurement and Reconstruction of a 3D Translucent Object Using Optical Filter

**Figure 4:**
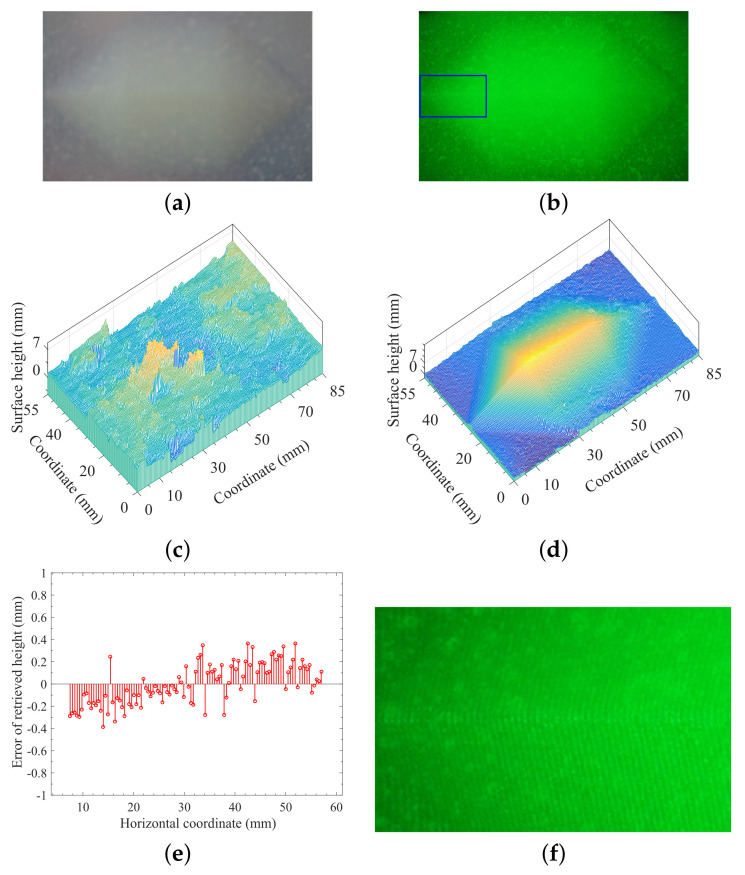
Measurement and reconstruction of the heptahedron-shape vulcanized silicone rubber illuminated by monochromatic sinusoidal fringe pattern at 532 nm under background illuminance *E* = 202 Lux. (**a**) Image without optical filtering. (**b**) Image with an optical bandpass filter centered at 532 nm with FWHM being 10 nm. (**c**) Reconstruction using the measurement of (**a**). (**d**) Reconstruction using the measurement of (**b**). (**e**) The absolute-error distribution of retrieved surface height on the cross section perpendicular to the ridge with actual values in accordance with data of (**d**). (**f**) The enlarged figure of the marked blue-rectangle region in (**b**).

As discussed above, the approach proposed in this work uses the projected laser-beam-based monochromatic fringe patterns, which allows the use of an optical bandpass filter for removing the effects of background illuminations. Figure 4 shows the measurement and reconstruction of a translucent heptahedron-shape object made of vulcanized silicone rubber under the background illuminance *E* = 202 Lux, while the spatial frequency of the projected fringe pattern was set to 2.2 lp/mm.

All other conditions and technical parameters, such as the output power of laser, the background illuminance *E*, the spatial frequency of the projected fringe pattern, and the adjustable parameters of the camera, were the same for the measurements in Figure 4a,b, except that the optical filter was used in the measurement of Figure 4b, but not in the case of Figure 4a. Taking the comparison between Figure 4a,b, the background illuminance with *E* = 202 Lux lowered the fringe contrast and increased the noised signal, and the distorted fringes on the surface of target being measured was almost submerged by the background illumination as indicated by Figure 4a, which resulted in the failed reconstruction as indicated in Figure 4c. However, for the same environmental and experimental conditions, the measurement of Figure 4b using optical filtering technique showed greatly-improved fringe contrast with much lower noise level, which ensured the accurate reconstruction of the target, as shown in Figure 4d. Obviously, the combination of monochromaticity of projected structured signal and optical filtering technique can ensure the high contrast of the projected fringe pattern, which is critically important for in situ profilometry with background illuminations. A problem has been noticed where it seems impossible to see the fringes in Figure 4b, which can be explained with the relatively high spatial frequency (2.2 lp/mm). However, the fringes on the object surface can clearly be seen when the image of Figure 4b is enlarged by a scale factor of 5 as shown in Figure 4f.

### 4.2. Measurement and Reconstruction of a 3D Translucent Object Using Optical Polarizer

**Figure 5:**
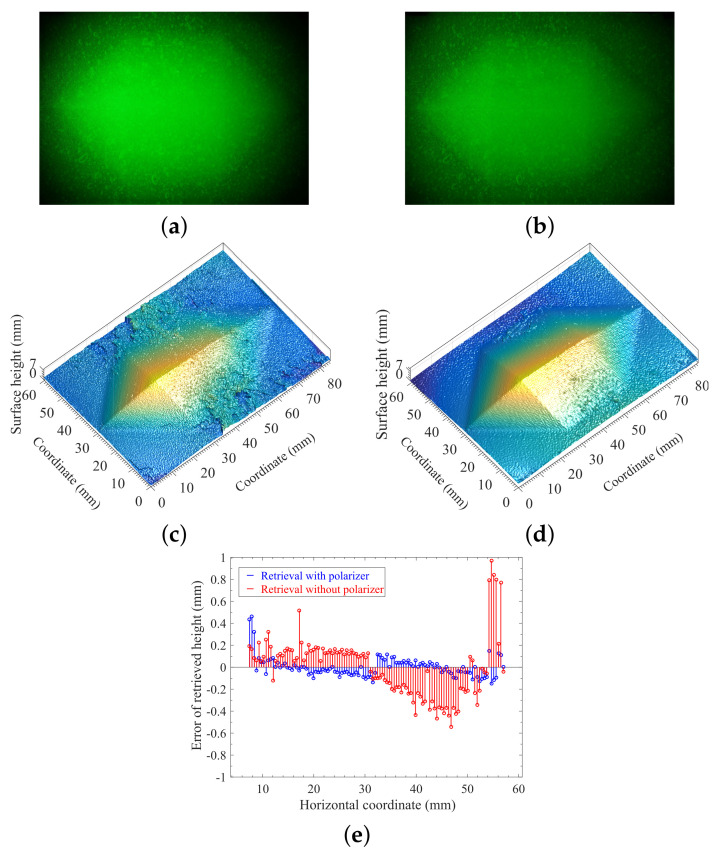
Measurement and reconstruction of the heptahedron-shape vulcanized silicone rubber illuminated by monochromatic sinusoidal fringe pattern at 532 nm without background illumination. (**a**) Image without an optical polarizer. (**b**) Image with an optical polarizer. (**c**) Reconstruction using the measurement of (**a**). (**d**) Reconstruction using the measurement of (**b**). (**e**) Comparing the absolute-error distribution of retrieved surface height on the cross section perpendicular to the ridge with actual values based on the data in (**c**,**d**).

For verifying the polarization of the reflected signal from the surface of the translucent object, we carried out the measurements as shown in Figure 5a,b with the following conditions: (i) The background illuminance E=0, thus the optical filter was not necessarily used. (ii) The spatial frequency of the projected fringe pattern was set to 4.0 lp/mm. The optical polarizer was not used in Figure 5a, while the measurement of Figure 5b was obtained by using optical polarizer via adjusting its direction of polarization.

The experimental condition of keeping the background illuminance being zero allowed us to study and assess the effect of multiply-scattered photons. The intensity of multiply-scattered photons basically depends on the thickness of the silicone rubber and the reflectivity of bottom surface. Due to the effect of multiply-scattered photons, the image of Figure 5a looked brighter and more blurred comparing to that of Figure 5b, which resulted in that the measurement of Figure 5b showed higher fringe contrast and yielded more accurate reconstruction of the object being observed as indicated in Figure 5d with comparing the result shown in Figure 5c.

Thus, the experimental results have proven the theoretical prediction discussed above that the effect of multiply-scattered photons can be removed using an optical polarizer, which is based on the physical basis that the projected monochromatic structured light and reflected signal from the surface of the translucent object are linearly polarized, and the signal component composing multiply-scattered photons is completely unpolarized.

### 4.3. Measurement and Reconstruction of 3D Translucent Objects under Different Background Illumination

Based on the experimental results of Section 4.1 and Section 4.2 above, we now present two sets of experimental measurements and reconstructions of two selected translucent objects under different background illumination, while both the optical filter and the polarizer are used simultaneously.

**Figure 6:**
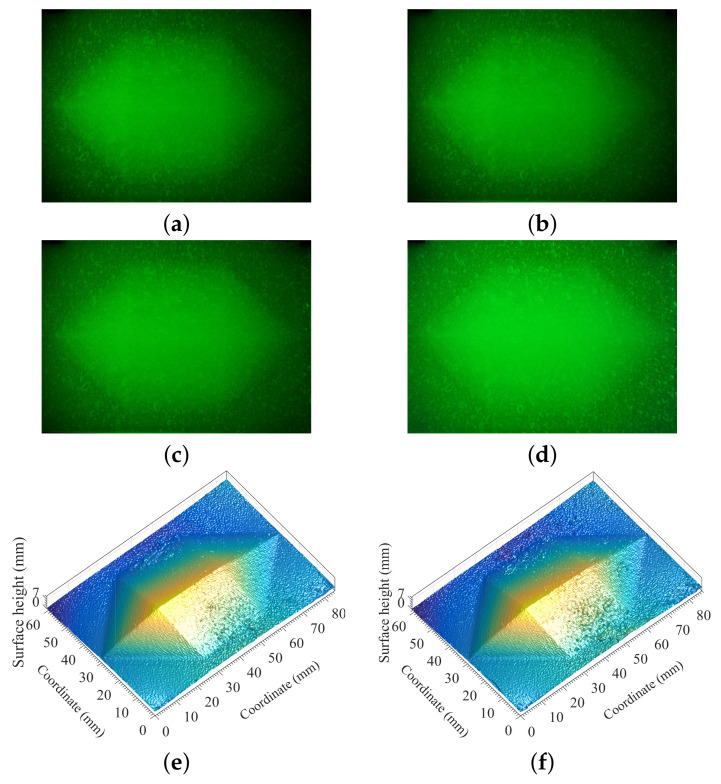

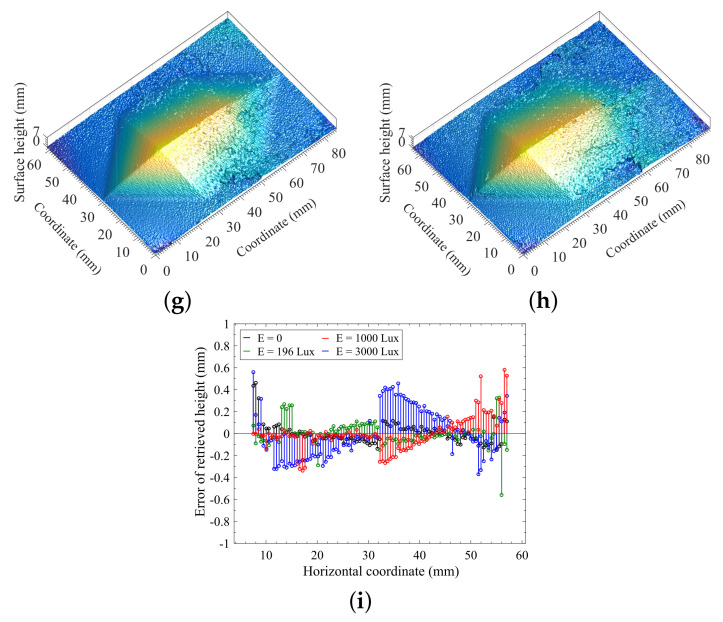
Measurement and reconstruction of the heptahedron-shape vulcanized silicone rubber illuminated by monochromatic sinusoidal fringe pattern at 532 nm under different background illuminance *E*. (**a**) Measurement of E=0. (**b**) Measurement of E=196 Lux. (**c**) Measurement of E=1000 Lux. (**d**) Measurement of E=3000 Lux. (**e**) Reconstruction using (**a**). (**f**) Reconstruction using (**b**). (**g**) Reconstruction using (**c**). (**h**) Reconstruction using (**d**). (**i**) Comparing the absolute-error distribution of retrieved surface height on the cross section perpendicular to the ridge with actual values based on the data in (**e**–**h**).

As shown in Figure 6, the first object was a translucent heptahedron-shape object made of vulcanized silicone, and the spatial frequency of the projected fringe pattern used in this part of the experiment was 4.0 lp/mm. We see from Figure 6a–d that the effects of background illumination and multiply-scattered photons can be mostly removed, but not completely.

The relatively high brightness of the image in Figure 6d of the measurement with background illuminance *E* = 3000 Lux indicates that the fringe contrast will be decreased as the background illuminance is increased. The results of Figure 6 might indicate that the accuracy of reconstruction using the measurement with background illuminance *E* = 196 Lux is the same as the case of *E* = 0 Lux, and the accuracy of reconstruction using the measurement with background illuminance *E* = 1000 Lux is basically satisfied. For the reconstruction using the measurement with background illuminance *E* = 3000 Lux as indicated in Figure 6h, the inaccuracy of reconstruction is mainly due to the decrease of the fringe contrast resulted from the very high background illuminance. Note that the fringe contrast of Figure 6h can be improved by increasing the output power of the laser source, which has been validated by preliminary experimental results.

**Figure 7:**
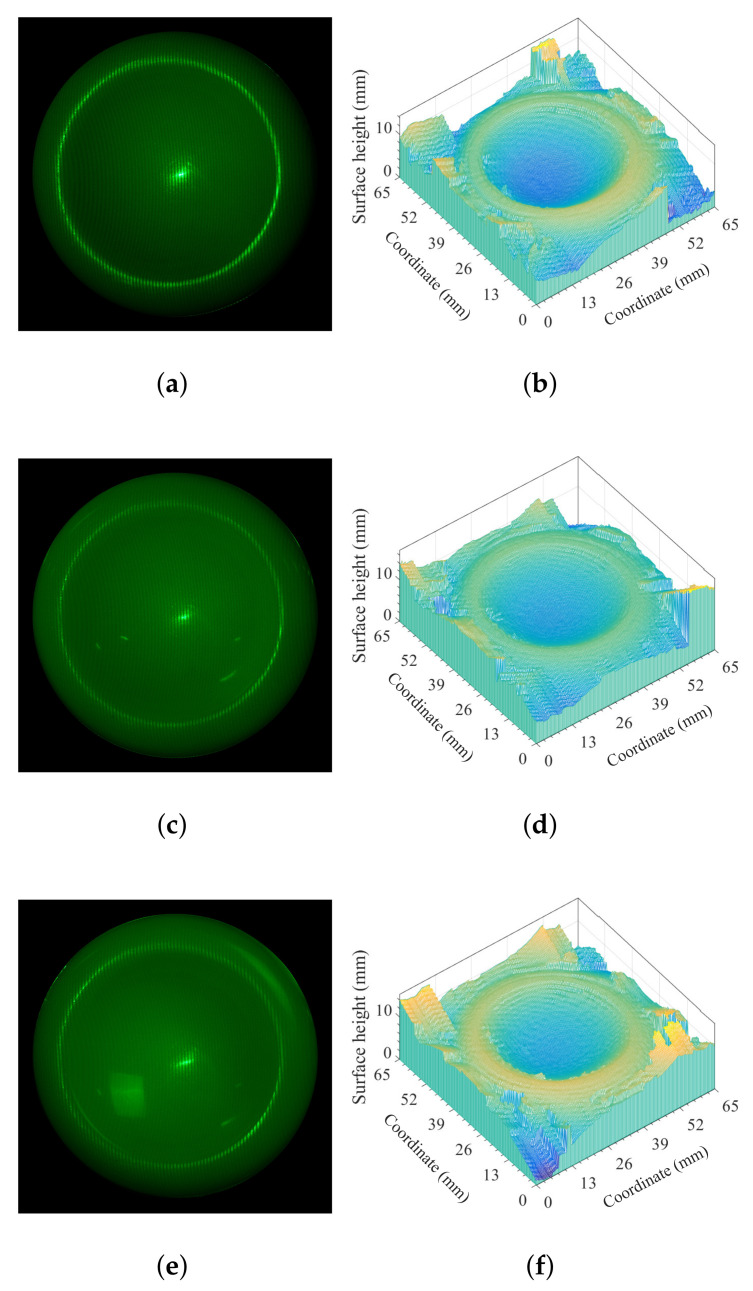
Measurement and reconstruction of the translucent plastic cap with different background illuminance *E*. (**a**) Measurement of E=0. (**b**) Reconstruction using (**a**). (**c**) Measurement of *E* = 50 Lux. (**d**) Reconstruction using (**c**). (**e**) Measurement of *E* = 180 Lux. (**f**) Reconstruction using (**e**).

For the second selected object, a translucent plastic cap made of polypropylene, the measurement and reconstruction were carried out with the spatial frequency of the projected fringe pattern being 1.7 lp/mm as shown in Figure 7.

The purpose of the experiment with translucent plastic cap made of polypropylene should be based on the consideration that the translucent plastic cap has different transparency, refractive index, shape, and surface smoothness. The spatial frequency of the projected fringe pattern used in Figure 7 was 1.7 lp/mm, and the intensity of the projected fringe pattern on the surface of the object was lower than that used in Figure 6.

Figure 7a,c,e were the measurements for background illuminance *E* = 0, 50, 180 Lux, respectively, while Figure 7b,d,f were the reconstruction using the measurements corresponding to Figure 7a,c,e, respectively. Generally speaking, the accuracy of reconstruction was accurate via comparing the reconstructed 3D shape with the geometrical parameters of the original object. Note that the we were unable to obtain accurate reconstruction if the background illuminance was increased to *E* = 1000 Lux as we had in Figure 6c, which might be mainly due to that the lower intensity of the projected fringe pattern on the surface of the object resulted in lower fringe contrast with *E* = 1000 Lux.

### 4.4. Analysis and Discussion of the Accuracy of Reconstruction

**Figure 8:**
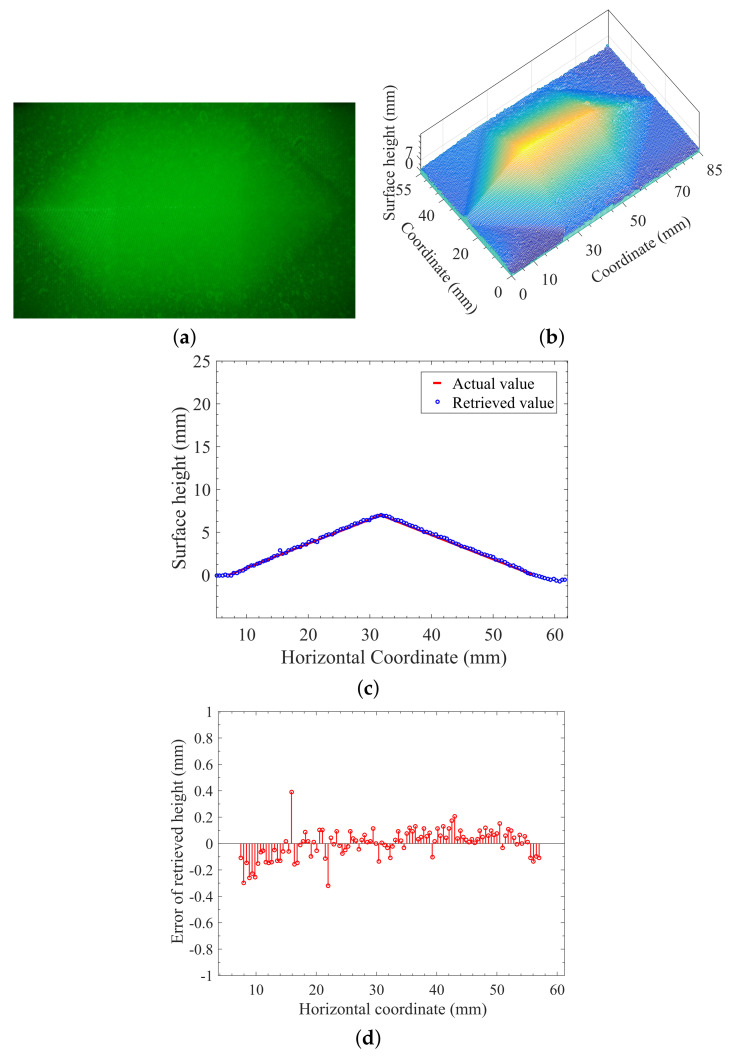
Measurement and reconstruction of the heptahedron-shape vulcanized silicone rubber illuminated by monochromatic sinusoidal fringe pattern at 532 nm under background illuminance *E* = 202 Lux. (**a**) Image of the illuminated target. (**b**) Reconstruction using the measurement of (**a**). (**c**) Comparing the retrieved height distribution on the cross section perpendicular to the ridge with actual values. (**d**) The absolute-error distribution of retrieved surface height in accordance with (**c**).

The analysis of the accuracy of reconstruction for the proposed method was based on the measurement and reconstruction of the designed heptahedron-shape target with given geometrical parameters. Figure 8a,b showed the measurement and reconstruction of the heptahedron-shape vulcanized silicone rubber, respectively, illuminated by monochromatic sinusoidal fringe pattern at 532 nm with the following experimental conditions: (i) The target being observed was illuminated by monochromatic sinusoidal fringe pattern at 532 nm. (ii) The spatial frequency of the projected fringe pattern is 2.2 lp/mm. (iii) The background illuminance E=202Lux. (iv) Both optical filter and polarizer were used for the measurement of Figure 8a.

The comparison of the retrieved height distribution on the cross section perpendicular to the ridge with actual values was described in the plot of Figure 8c, and the absolute-error distribution of retrieved surface height in accordance with Figure 8c is given in Figure 8d. The accuracy of reconstruction can be obtained using the comparison between the retrieved height distribution on the cross section perpendicular to the ridge and the actual geometrical parameters of the target being observed. We should point out that the individual point of largest deviation in Figure 8d was due to the little hump or hole on the surface of object, since the translucent heptahedron-shape object made of vulcanized silicone in our lab did not have a perfect geometrical shape as designed.

To quantitatively evaluate difference between known and calculated geometry of the object being observed, we employ the root mean square error (RMSE) for telling the accuracy of reconstruction. The lower the RMSE, the better the accuracy of the reconstruction. The calculated RMSE using the data of Figure 4e, Figure 5e, Figure 6i and Figure 8d are listed in Table 1 below. From Table 1, we see that RMSE = 0.09–0.11 (mm) can be obtained for usual indoor background illumination when both optical filter and polarizer are used in our developed method. However, when the background illumination is increased such as the case of Figure 6c,d, RMSE = 0.16–0.23 (mm) may be acceptable for many applications.

The results of Figure 8 and Table 1 may indicate that an accurate reconstruction can be obtained using 3D surface-shape measurement of the translucent object with the combination of laser-beam-based structured light and polarization technique, and the optical filtering technique should also be included and works well if the background illumination exists.

The success of the proposed method for 3D measurement and reconstruction of translucent objects is definitely based on the following items: (i) high-contrast and high-quality sinusoidal fringe projection, (ii) polarization technique, and (iii) optical filtering technique. Note that the projected structured light with the optical wave from laser source is monochromatic and generally polarized, thus both the optical filtering and polarization techniques can be used for measurement and image processing as we have proposed in our investigation. However, since the optical signal generated by a DLP-based projector is non-monochromatic and unpolarized, it is impossible to extend our approach to the methods and applications that are based on the use of structured light generated by a DLP projector.

It should be noted that the accuracy of 3D measurement and reconstruction of translucent objects is basically determined by the intensity, contrast, and spatial frequency of the projected fringes and the background illumination. The optical parameters including the spatial frequency of projected fringes should be optimized according to the measuring conditions. The intensity and contrast of the projected fringes are related to the output power of the laser beam and the background illumination, while the possibly higher output power of the laser beam might be necessary when the background illuminance is higher.

## 5. Conclusions

Based on theoretical analyses and experimental results of this study, we have demonstrated that reliable measurements and accurate reconstructions can be obtained for determining 3D surface shapes of translucent objects being observed with the combination of laser-beam-based fringe projection, the polarization technique, and the optical filtering technique. The rigorous theoretical analyses presented in this study have described the formation, propagation, and physical feature of the generated sinusoidal signal by the designed optical system, the reflection and transmission of the projected monochromatic fringe pattern on the surface of the translucent object, and the formation and the separation of the direct-reflection and the global components of the surface radiance of the observed object. The designed novel sinusoidal optical signal generator can generate monochromatic, high contrast, truly sinusoidal, and linearly polarized fringe patterns, which is the critical basis for this study. The polarization technique should be emphasized in the developed method of this work for eliminating the effect of multiply-scattered photons under the condition that the projected fringe pattern is generated using designed sinusoidal optical signal generator. The optical filtering technique is important and necessary for removing the effect of the background illumination since the projected fringe pattern is a monochromatic signal. It should be noted that the optical system used in this work is portable and stable, and can be used for outdoor or in situ measurement and reconstruction of translucent objects. The results of the reliable measurement and accurate reconstruction of the heptahedron-shape vulcanized silicone rubber illuminated by monochromatic sinusoidal fringe pattern at 532 nm under the background illuminance *E* = 0∼1000 Lux shown in Figure 6 as well as the error analysis presented in Figure 8 are capable of proving the success of the developed approach of this work. The expected applications of the developed method and result of this work may include accurate in situ shape determination of any 3D translucent objects and related target recognition under general environmental conditions.

## Figures and Tables

**Table 1:** Comparison of RMSE of different reconstructions.

No. of Figure	Color of Data	RMSE (mm)
Figure 4e	red	0.18
Figure 5e	blue	0.09
	red	0.28
Figure 6i	black	0.10
	green	0.11
	red	0.16
	blue	0.23
Figure 8d	red	0.11

## Data Availability

The data presented in this study are available on request from the corresponding author.
